# Mental Health, Emotional Regulation, and Psychosocial Work Factors Among Scientific Researchers: A Cross-Sectional Study from Paraguay

**DOI:** 10.3390/brainsci15010065

**Published:** 2025-01-13

**Authors:** Julio Torales, Anthon Torres-Romero, Iván Barrios, Marcelo O’Higgins, Tomás Caycho-Rodríguez, João Mauricio Castaldelli-Maia, Antonio Ventriglio

**Affiliations:** 1 Facultad de Ciencias Médicas, Cátedra de Psicología Médica, Universidad Nacional de Asunción, San Lorenzo 111421, Paraguay; juliotorales@gmail.com (J.T.); antiel.ts.ro@gmail.com (A.T.-R.); marcelo.g.ohiggins@gmail.com (M.O.); 2 Facultad de Ciencias de la Salud, Universidad Sudamericana, Salto del Guairá 140101, Paraguay; ivanjuan2013@gmail.com; 3 Facultad de Ciencias Médicas, Filial Santa Rosa del Aguaray, Cátedra de Bioestadística, Universidad Nacional de Asunción, Santa Rosa del Aguaray 021801, Paraguay; 4 Facultad de Psicología, Universidad Científica del Sur, Lima 15067, Peru; tcaycho@cientifica.edu.pe; 5 Department of Neuroscience, Fundação do ABC, Santo André 09070870, SP, Brazil; jmcmaia2@gmail.com; 6 Department of Psychiatry, University of São Paulo, São Paulo 05403903, SP, Brazil; 7 Department of Clinical and Experimental Medicine, University of Foggia, Viale Pinto n. 1, 71121 Foggia, Italy

**Keywords:** anxiety, depression, psychological stress, work-related stress, emotional regulation

## Abstract

Background: This study examined the prevalence of mental health issues among Paraguayan researchers and their relationships with emotional regulation and psychosocial factors. Methods: A cross-sectional survey of 235 researchers was conducted using validated instruments: the Depression, Anxiety, and Stress Scale (DASS-21); the Job Content Questionnaire (JCQ); and the Difficulties in Emotion Regulation Scale (DERS). Sociodemographic, academic, and behavioral variables were also analyzed. Results: Findings revealed significant rates of depression (26.4%), anxiety (30.6%), and stress (32.3%), with female researchers reporting nearly twice the rates of anxiety and stress compared to males. Researchers with doctoral degrees exhibited lower anxiety levels, emphasizing the protective role of advanced academic qualifications. Conversely, younger and early-career researchers were more vulnerable to psychological distress. High job demands and emotional dysregulation were strongly associated with poorer mental health outcomes, while hazardous alcohol consumption and low physical activity further exacerbated risks. Conclusions: These findings highlight the urgent need for institutional reforms to prioritize mental health and well-being in academic environments. By advancing the understanding of occupational health in resource-limited settings, this study provides actionable recommendations to improve the working conditions and mental health of researchers in Paraguay and beyond.

## 1. Introduction

The mental health and well-being of scientific researchers have recently gained attention as they are highly vulnerable to workplace stress due to demanding job requirements [1]. They encounter heavy workloads, academic pressures, and social expectations that adversely affect their psychological health and professional performance. Examining the prevalence of mental health issues and their link to working conditions may help create targeted interventions to enhance researchers’ quality of life and productivity [2].

Worldwide, workplace mental health is becoming a growing concern [3]. Owing to the nature of their work, scientific researchers face high stress levels and constant demands to publish, secure funding, and meet strict deadlines [4,5]. These pressures can lead to mental health challenges such as depression, anxiety, and chronic stress, impacting both individual and scientific progress. Additionally, prolonged stress without sufficient resources or support can lead to burnout, which is now recognized as a significant occupational hazard in academic settings.

A balance between job demands and job control is critical for researchers’ mental health [6]. Karasek’s demand–control model, despite being established over 45 years ago, remains a cornerstone in occupational health research due to its robust validity and reliability across various professional settings. This model posits that high job demands paired with low work control increase the risk of mental disorders [7]. Its adaptability to diverse contexts, including academic research environments, and its cultural validation for use in Latin America [8] further support its relevance in understanding the psychosocial work factors affecting researchers in Paraguay. Furthermore, emotional regulation, which involves managing and responding to emotions, and personal traits, such as consistency and perseverance, significantly affect coping with work-related stress [9].

Psychological distress, defined as a state of emotional discomfort or suffering characterized by symptoms of anxiety, depression, and stress [10], is notably prevalent among researchers, with rates ranging from 13.4% among all researchers [11] to 40% among early-career researchers [12]. A study by Hill et al. [11] reported that 13.4% of researchers met the threshold for severe psychological distress, a rate significantly higher in early-career researchers compared to participants from other career stages. Among those who answered questions on mental health diagnoses and suicidal ideation, 54% reported a lifetime mental health diagnosis, and 23.7% reported suicidal ideation since the start of their academic career [11]. Similarly, Milicev et al. [12] found high levels of ill mental health and low well-being among postgraduate researchers in the UK, with poorer outcomes linked to factors such as gender, sexual identity, perfectionism, workaholism, and extended study durations [12]. Despite these findings, there is a lack of reliable data on the mental health of researchers in resource-constrained regions like Paraguay, revealing a significant knowledge gap. This study specifically addresses this gap by focusing on Paraguayan researchers, a population that faces unique systemic and cultural challenges thereby contributing new insights to the global literature.

### 1.1. Contextualization of the Study

Scientific researchers in Paraguay face distinct challenges, including systemic underfunding, limited institutional resources, and high academic demands. These challenges are compounded by pressures to publish, secure grants, and manage administrative responsibilities, creating a uniquely stressful environment. Unlike researchers in higher-income countries, Paraguayan researchers often operate with fewer resources and less institutional support, making them particularly vulnerable to psychological distress [13].

This study is especially important in the Paraguayan context, where little is known about how systemic challenges impact researchers’ mental health. By addressing this gap, the research aims to provide insights that inform targeted interventions and policies tailored to resource-limited environments. Additionally, it contributes to global occupational mental health literature by offering data from an underrepresented academic context.

### 1.2. Objectives and Theoretical Underpinning

Building on the contextual challenges outlined above, this study aims to assess the prevalence of mental health issues among Paraguayan researchers and examine their relationships with emotional regulation and psychosocial factors such as job demands, job control, and social support. Specifically, it investigates the following: (a) the prevalence of psychological distress (depression, anxiety, and stress) among Paraguayan researchers, and (b) the relationship between psychosocial work factors, emotional regulation, and mental health outcomes, while considering the influence of sociodemographic, academic, behavioral, and health-related variables.

Grounded in Karasek’s demand–control model and theories of emotional regulation [7,8], this research seeks to provide a comprehensive understanding of the factors influencing mental health in academic settings. These findings aim to inform interventions that promote healthier work environments for researchers in Paraguay and beyond.

## 2. Methods

### 2.1. Designing and Sampling

This observational, descriptive, and cross-sectional study employed a prospective design, which allows for the collection of data from participants at a single point in time while planning data collection to address specific research objectives [14]. This approach is suitable for examining relationships between variables, such as psychosocial work factors and mental health outcomes, within a defined population. However, the cross-sectional design limits causal inferences, which is acknowledged as a methodological limitation in this study.

A non-probabilistic convenience sampling method was used to select participants. This approach was justified due to the accessibility of researchers listed in the open-access database of the Paraguay National System of Researchers (SISNI) from the National Council of Science and Technology (CONACYT) [15]. While this method ensured participation across the five scientific areas defined by CONACYT—(1) Agricultural and Veterinary Sciences; (2) Engineering and Technology, including Informatics and Mathematics; (3) Social Sciences and Humanities; (4) Medical and Health Sciences; and (5) Natural Sciences, including Physics, Chemistry, and Biology—it may limit the generalizability of the findings, a limitation addressed in the discussion section.

Inclusion criteria required participants to be registered in SISNI, actively involved in research activities within one of the five scientific areas, and to provide informed consent to participate in the study. Exclusion criteria included incomplete survey responses and researchers no longer actively engaged in research activities. Incomplete or inconsistent data were carefully reviewed, and cases with missing values in key variables were excluded from specific analyses. Outliers were identified using standardized statistical methods and excluded only if they significantly distorted results, ensuring data integrity.

All participants received detailed information about the study’s purpose, data protection measures, and their rights, including the voluntary nature of participation and the option to withdraw at any time without consequences. Data protection and confidentiality were ensured in accordance with national regulations, and personal identifiers were removed to anonymize responses.

To address potential common method bias, the following several strategies were employed: (a) ensuring anonymity and confidentiality to minimize social desirability bias; (b) structuring the online survey to include well-established, validated scales for measuring key constructs; and (c) using diverse question formats and reverse-coded items to reduce the risk of response patterns.

Data were collected from August to October 2024 through an online survey distributed via email addresses available in the CONACYT database.

### 2.2. Sample Size

The sample size was determined using the Epidat 4.2 epidemiological package (Pan American Health Organization, Junta de Galicia, and CES University of Colombia). Based on a population of 696 active researchers registered in SISNI through CONACYT and an anticipated psychological distress prevalence of 13.4% [11], with a 95% confidence level and 5% precision, the minimum required sample size was 143 participants [16]. Ultimately, 235 researchers participated in the study.

### 2.3. Measures

The selection of measures in this study was guided by a theoretical framework emphasizing the multifaceted nature of mental health and its interplay with work demands, emotional regulation, and addictive behaviors. Drawing upon the framework proposed by Di Giacomo et al. [17], this study highlights the critical interplay between psychological dimensions such as depression, anxiety, stress, emotional regulation, consistency, and perseverance in academic settings. Depression, anxiety, and stress were included as key indicators of mental health, given their high prevalence among high stress populations such as medical students. Work demands, control, and social support, derived from the Job Demand–Control–Support model, capture the psychosocial stressors inherent in academic and professional settings. Emotional regulation was assessed due to its pivotal role in mitigating stress and maintaining psychological well-being, while addictive behaviors such as alcohol and substance use were evaluated because of their potential as maladaptive coping mechanisms in high stress environments. Together, these measures provide a robust basis for understanding the mental health challenges and coping mechanisms of the target population.

Sociodemographic variables: Participants provided information on their gender (male or female), age, residence (Asunción, Central, rest of the country), marital status (single, married/partnered, divorced/separated, widowed), and parenthood status (yes, no, number of children).

The academic variables included academic background, the highest educational level (bachelor’s, master’s, doctorate), SISNI categorization (Beginner, Level I, II, III per national research system), scientific field (Social Sciences and Humanities; Medical and Health Sciences; Natural Sciences like Physics, Chemistry, Biology; Agricultural and Veterinary Sciences; Engineering and Technology, including Computer Science and Mathematics), and primary institution type (public or private).

Behavioral and health variables: Participants reported lifestyle habits such as physical activity (at least 150 min of moderate-intensity or 75 min of high-intensity exercise per week, per the Ministry of Public Health and Social Welfare guidelines) [18], alcohol and substance use, tobacco use, electronic cigarette use, and illicit drug use (yes or no). They also provided details about their health and mental well-being, including their mental health conditions (yes, no, specify) and current treatment (pharmacotherapy, psychotherapy, or both).

Assessments: According to suggestions by Di Giacomo et al. [17], participants’ mental health was measured using the Depression, Anxiety, and Stress Scale (DASS-21). Job demands, control, and social support were evaluated using the Job Content Questionnaire (JCQ); emotional regulation using the Difficulties in Emotion Regulation Scale (DERS); and perseverance and interest consistency using the Short Grit Scale. Hazardous alcohol and substance use were assessed using MULTICAGE CAD-4 (Screening questionnaire for impulse control disorders and addictions at the Drug Dependency Care Center (CAD) 4 San Blas, part of the Addiction Institute of Madrid Salud, Spain).

DASS-21 is a self-reporting measure of depression, anxiety, and stress, and contains seven items per subscale. The depression scale assesses dysphoria, hopelessness, devaluation of life, self-deprecation, lack of interest, anhedonia, and inertia. The anxiety scale evaluates autonomic arousal, musculoskeletal effects, situational anxiety, and anxious affect, whereas the stress scale measures chronic nonspecific arousal, difficulty relaxing, nervous arousal, and tendencies to become upset, irritable, and impatient [19]. Participants report their experiences over the past week using a 4-point severity/frequency scale. The scores for each scale are summed, and a general emotional symptom score is obtained by summing all item scores, with higher scores indicating higher levels of symptoms. Cutoff points are provided for each scale [20]. The Spanish version by Ruiz et al. [20], used in this study, exhibits good psychometric properties, with Cronbach’s α ranging from 0.80 to 0.95 for the total scale and subscales. Additionally, the general factor of DASS-21 accounts for more than 70% of the variance [20].

MULTICAGE CAD-4, developed in Spanish by the Drug Dependency Care Center (CAD) 4 San Blas of the Addiction Institute of Madrid Salud, consists of 32 items designed to assess various addictions and disorders, including alcohol abuse/dependence, pathological gambling, substance addiction, eating disorders, Internet addiction, video game addiction, compulsive spending, and sex addiction [21]. Each issue is assessed using four questions based on CAGE (Cut Down, Annoyed, Guilty, Eye-Opener) scheme [22], focusing on self-perception, others’ perceptions, guilt, and withdrawal or loss of control. The self-administered questionnaire employs a Yes/No dichotomous scale, with the number of affirmative responses indicating the severity of the problem. Two or more affirmative responses per subscale suggest problematic behaviors. The full scale and its subscales demonstrate good psychometric properties, with Cronbach’s α exceeding 0.7 for all subscales. Additionally, the eight components identified in the exploratory factor analysis accounted for 63.8% of the total variance, reflecting strong reliability and validity of the scale [21].

Karasek’s Job Content Questionnaire (JCQ) was used to assess the psychosocial aspects of work, including job demands, job control, and social support [23]. A 21-item Latin American-validated version of JCQ was used, featuring three subscales with Likert-format items: strongly disagree, disagree, agree, and strongly agree [24]. Higher scores indicate more demanding jobs, greater autonomy, and more perceived support from colleagues and supervisors for job demands, job control, and social support subscales, respectively. The subscales showed good internal consistency, with Cronbach’s alpha values of 0.87 for job demands, 0.80 for job control, and 0.91 for social support. The variance explained by these subscales was 27.41%, 45.73%, and 7.18%, respectively [24].

The 18-item Difficulties in Emotion Regulation Scale (DERS) assesses challenges in emotional regulation across six dimensions: awareness (items 1, 4, 6), clarity (items 2, 3, 5), goals (items 8, 12, 15), impulsivity (items 9, 16, 18), non-acceptance (items 7, 13, 14), and strategies (items 10, 11, 17) [25]. Participants may rate the frequency of difficulties on a scale ranging from 1 = almost never (0–10%) to 5 = almost always (91–100%). Scores are totaled by summing the items, with items 1, 4, and 6 reverse scored. Higher scores denote greater emotional dysregulation. The Spanish version by Navarro Carrascal et al. [26], used in this study, exhibits good psychometric properties, with a Cronbach’s α of 0.83 for the total scale. Additionally, the scale demonstrates excellent structural validity, as indicated by all fit indices [26].

The Original Grit Scale (Grit-O) developed by Duckworth et al. [27] features 12 items rated on a Likert scale from 1 to 5, based on participant agreement. In 2019, a shortened version of the Grit Scale Short (Grit-S) [28] was introduced, and a Spanish adaptation for the Latin American context was utilized in this study, demonstrating a Cronbach’s alpha of 0.834 and a total variance of 43.641% [29]. The test includes the following two dimensions:

- Consistency: This assesses the maintenance of long-term goals through four negatively worded items, where higher agreement indicates lower consistency, necessitating score reversal during the analysis.
- Perseverance: This measures sustained effort despite obstacles via four positively worded items rated from 1 to 5, with higher scores indicating greater perseverance [29].

### 2.4. Data Analysis

Jamovi and RStudio facilitated data management and analysis. Descriptive statistics included frequency tables for categorical variables and measures of central tendency and dispersion for numerical variables. The mean DASS-21 scores for depression, anxiety, and stress were compared using a two-tailed *t*-test, with a significance level of *p* < 0.05. DASS-21 scores were categorized into ‘normal’, ‘mild’, ‘moderate’, ‘severe’, and ‘extremely severe’ according to the DASS-21 manual, and these groups were compared using Pearson’s chi-square test at a 5% significance level. Pearson’s correlation test was employed to evaluate the relationships between numerical variables, such as age, years of research experience, and emotional regulation scores. The direction of significant associations was determined using the Odds Ratio (OR) with a 95% confidence interval.

### 2.5. Ethical Considerations

This study was approved by the Department of Medical Psychology at the School of Medical Sciences, National University of Asunción, Paraguay (Reference 008-008-2024). The study adhered to the principles of confidentiality, equality, and fairness outlined in the Declaration of Helsinki. The participants provided informed consent to participate in the survey, and those who wished to receive feedback from the investigation were invited to contact a designated email for their responses or specific suggestions.

## 3. Results

**Figure 1:**
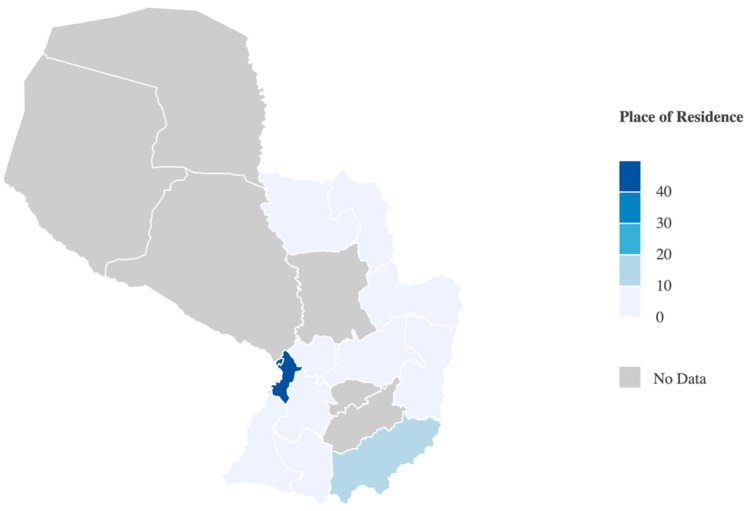
Participants’ places of residence (N = 235).

This study assessed 235 researchers participating in the Paraguay SISNI program. Of these participants, 53.6% were women and 62.6% were married or partnered. The average age was 44.54 ± 10.51 years (Table 1). The researchers were from the capital city (Asunción) and 11 of the 17 Paraguay departments, with the majority residing in the Central department (40.0%) (Figure 1).

Table 2 reveals that 31.9% of the participants were performing research in the fields of Social Sciences and Humanities. In terms of academic qualifications, 48.5% held doctorates and 45.5% held master’s degrees. Most researchers were employed at public institutions (75.3%) and were novices at SISNI (50.2%).

Concerning behavioral and health variables, 40.9% of researchers did not meet the recommended physical activity levels (14), engaging in less than 150 min weekly, and 28.5% did not engage in any physical activity. Regarding habits, 47.2% of the participants consumed alcohol, 3.4% used substances, 4.7% smoked tobacco, and 1.3% used e-cigarettes. Furthermore, 5.1% reported mental health conditions, predominantly anxiety disorders, with psychotherapy being the most common treatment (50%) (Table 3).

According to DASS-21 scores, 26.4% of the researchers tested positive for depression (subscale score ≥ 5), with 3.4% showing extremely severe depression (subscale score ≥ 14). Anxiety was reported by 30.6% of the participants (subscale score ≥ 3), and 6.4% experienced extremely severe anxiety (subscale score ≥ 10). Regarding stress, 32.3% reported some level of stress (subscale score ≥ 7), and 2.1% reported extremely severe stress (subscale score ≥ 17). Additionally, 8.9% screened positive for alcohol problems (MULTICAGE CAD-4 alcohol subscale score ≥ 2) and 0.8% for drug problems (MULTICAGE CAD-4 drug subscale score ≥ 2) (Table 4).

The mean job demands score, as per JCQ, was 14.99 (SD = 2.915), job control was 18.14 (SD = 2.816), and social support was 27.65 (SD = 5.018). Emotional dysregulation, as measured by DERS, reported a mean of 38.71 (SD = 12.988), highlighting difficulties in emotional clarity and impulse control. The Grit-S scale scores for consistency and perseverance were 10.11 (SD = 3.876) and 15.86 (SD = 3.380), respectively. These findings indicate high job demands and emotional dysregulation among participants with lower long-term goal consistency and greater perseverance.

Pearson’s chi-square tests analyzed the relationships between sociodemographic variables and behavioral habits of researchers in the Paraguayan SISNI program. The study identified a significant association between gender and alcohol consumption [χ^2^(2) = 16.551, *p* < 0.001]. Male researchers were almost twice as likely to report an alcohol use disorder, with an OR of 1.997 (0.795–5.017).

A significant relationship between anxiety and gender was observed [χ^2^(1) = 5.676, *p* = 0.017)], with female researchers reporting an OR of 1.999 (1.126–3.550), suggesting that they are nearly twice as likely to experience anxiety. Similarly, gender was significantly associated with significant stress [χ^2^(1) = 4.111, *p* = 0.043)], with female researchers showing an OR of 1.780 (1.017–3.117), indicating a higher likelihood of experiencing stress.

The Pearson correlation test revealed negative correlations between age and depression scores (r = −0.208, *p* = 0.001), anxiety (r = −0.214, *p* = 0.001), and stress (r = −0.223, *p* = 0.001), as well as with DERS dimensions, such as clarity (r = −0.262, *p* < 0.001), non-acceptance (r = −0.189, *p* = 0.004), and strategies (r = −0.176, *p* = 0.007). This indicates that younger researchers reported higher levels of anxiety, depression, and emotional regulation difficulties than older researchers.

Years of research experience showed a positive correlation with age and negative correlations with depression (r = −0.095, *p* = 0.147), anxiety (r = −0.108, *p* = 0.097), and stress (r = −0.120, *p* = 0.066), though these were not statistically significant. The analysis revealed a significant negative correlation with emotional regulation difficulties (r = −0.143, *p* = 0.028) and positive correlations with the consistency and perseverance dimensions of the Grit-S scale (r = 0.197, *p* = 0.002; r = 0.169, *p* = 0.010). These results indicate that more experienced researchers tend to exhibit better emotional control and greater persistence.

Pearson’s chi-square test revealed a significant association between anxiety and the educational level of participants (χ^2^ = 12.724, *p* = 0.002). However, the analysis did not reveal significant associations between educational level and depression (χ^2^ = 1.747, *p* = 0.417) or stress (χ^2^ = 3.868, *p* = 0.145). Disaggregated analysis by educational level revealed that researchers with doctoral degrees had a significantly lower prevalence of anxiety (χ^2^ = 7.901, *p* = 0.005), while no statistically significant associations were identified for researchers with master’s or bachelor’s degrees across any of the evaluated domains (*p* > 0.05). Risk estimation using OR indicated that holding a doctoral degree may act as a protective factor against anxiety. For the total sample, the OR was 0.442 (95% CI: 0.249–0.786), suggesting that researchers with doctoral degrees are less likely to experience anxiety compared to those without this level of academic attainment. Subgroup analysis revealed that among researchers with doctoral degrees, the OR was 0.636 (95% CI: 0.450–0.899), further supporting the protective effect of this academic level. In contrast, among researchers without doctoral degrees, the OR was 1.438 (95% CI: 1.133–1.825), indicating a higher relative risk of anxiety compared to those with doctoral degrees. This value aligns with the mathematical inverse of the general OR, reinforcing the interpretation that not holding a doctoral degree is associated with an increased risk of anxiety.

The MULTICAGE CAD-4 alcohol subscale scores were significantly correlated with depression (r = 0.250, *p* = 0.008), linking alcohol consumption to higher depression levels. It was also positively correlated with various DERS dimensions, indicating that alcohol use is associated with increased emotional regulation difficulties.

Depression and anxiety showed a strong correlation (r = 0.692, *p* < 0.001), reflecting their comorbidity. Both were significantly linked to stress (depression: r = 0.727, *p* = 0.0001; anxiety: r = 0.728, *p* < 0.001), job demands (depression: r = 0.211, *p* = 0.001; anxiety: r = 0.236, *p* < 0.001), and emotional regulation difficulties (depression: r = 0.591, *p* < 0.001; anxiety: r = 0.498, *p* < 0.001). This finding suggests that researchers facing higher job demands and lower social support reported high levels of depression, anxiety, and stress.

The *t*-test analysis indicated significant differences between men and women in several variables. Women reported higher anxiety levels than men, with average scores of 3.71 and 2.23, respectively. The results [t(233) = 3.026, *p* = 0.003] confirmed that women experienced significantly higher levels of anxiety.

A notable gender difference in drug use was identified, with men scoring an average of 1.75 on the MULTICAGE CAD-4 drug use subscale, compared to women’s average score of 0. This result was statistically significant [t(6) = 3.656, *p* = 0.011], indicating a higher frequency of drug use among men, potentially related to stress factors or differing coping mechanisms between genders.

Men reported higher job demands than women, with an average of 15.45 compared to 14.45 for women (t(233) = 2.664, *p* = 0.008). This suggests that men perceive significantly greater work pressure, potentially affecting their well-being and productivity.

Participants were also divided based on their emotional regulation difficulties (the emotional awareness subscale of DERS). Group 1 (Beginner/Level I in SISNI) reported a significantly higher mean score than Group 2 (Level II/Level III) (M = 8.00 ± 3.301 vs. M = 6.68 ± 3.111), indicating more difficulty in emotional awareness (t(233) = 2.270, *p* = 0.024). Additionally, the emotional clarity subscale showed a significant difference (t(233) = 2.425, *p* = 0.016), with Group 1 scoring higher (M = 4.98 ± 2.792) than Group 2 (M = 3.84 ± 1.685), suggesting that Group 1 struggled more with understanding their emotions.

Regarding consistency as measured by the Grit-S scale, Group 1 reported a significantly higher average score than Group 2 (M = 10.37 ± 3.857 vs. M = 8.79 ± 3.750), with a significant difference (t(233) = 2.324, *p* = 0.021), suggesting greater long-term effort consistency in Group 1. Conversely, in the perseverance subscale of Grit, Group 2 scored higher (M = 17.03 ± 2.843 vs. M = 15.64 ± 3.436 in Group 1), with a significant difference (t(233) = −2.337, *p* = 0.020), indicating greater perseverance in Group 2.

Perceived social support, evaluated using JCQ, differed significantly between the groups (t(233) = 2.446, *p* = 0.036). Researchers in the capital/central region averaged 28.05 ± 4.708, indicating higher social support compared to those in the country’s interior, who averaged 26.08 ± 5.874. This implies that individuals in the capital/central region reported stronger support systems, possibly because of better work or social integration.

Conversely, variables related to depression, anxiety, stress, and other aspects of emotional regulation (e.g., emotional clarity, impulse control, goals, non-acceptance, and strategies) did not show significant differences between the groups. The analysis did not detect significant differences in the alcohol subscale scores of MULTICAGE CAD-4 or in perceived job demands and job control between groups.

## 4. Discussion

This study represents the first effort to examine the prevalence of mental health issues among Paraguayan researchers and to analyze their relationships with emotional regulation and psychosocial factors. It provides critical insights into the interplay of sociodemographic, academic, and behavioral variables with mental health outcomes in an underrepresented academic population.

Our results revealed that 53.6% of the participants in the study were women, suggesting progress toward gender equity in academia, an area where women have historically been underrepresented [30]. However, this proportion raises important questions about whether gender equity is also reflected in leadership roles, funding opportunities, and professional development. Future studies should explore these dimensions to assess whether the observed representation translates into equitable access to career advancements and resources for women researchers.

Our results reported significant rates of depression (26.4%), anxiety (30.6%), and stress (32.3%) among Paraguayan researchers. These findings align with prior research indicating that academic researchers are particularly vulnerable to mental health challenges due to factors such as publication pressure, job instability, and the constant need to secure research funding [11,12,31,32,33]. The association between gender and mental health outcomes was particularly notable. Female researchers in this study were nearly twice as likely to report anxiety and stress compared to their male counterparts. This finding mirrors global trends and may stem from sociocultural expectations, gender roles, and the added burden of balancing professional and personal responsibilities [34,35,36,37]. Interestingly, no significant gender differences were observed in depression rates. This divergence warrants further investigation to understand the nuanced ways in which gender impacts specific mental health conditions. Additionally, the perception of inequality in professional opportunities and recognition may amplify stress and anxiety among female researchers [36,37].

Our findings also highlight the protective role of advanced academic qualifications against anxiety. Researchers with doctoral degrees exhibited significantly lower anxiety levels, suggesting a buffering effect. This may reflect enhanced coping mechanisms, professional stability, and institutional support associated with higher academic status. By contrast, researchers without doctoral degrees faced greater uncertainty regarding career progression and resource access, increasing their vulnerability to anxiety [38,39]. Notably, no significant associations were observed between educational level and depression or stress, indicating that the protective factors linked to doctoral qualifications may be more specific to anxiety.

The high job demands and emotional dysregulation observed among participants underscore the critical need for institutional interventions. High job demands often lead to emotional overload, which, when coupled with limited resources and support, exacerbates emotional dysregulation and reduces productivity [40,41,42]. These challenges were particularly evident in researchers with less work experience, who reported higher levels of depression, anxiety, and stress compared to their senior counterparts. This generational disparity may stem from early-career pressures, including establishing academic credibility and securing stable employment [43,44]. Mentorship programs and targeted mental health initiatives for early-career researchers could mitigate these vulnerabilities [45].

A concerning finding was the low engagement in physical activity among researchers, with 40.9% failing to meet recommended levels and 28.5% reporting no regular exercise. Physical inactivity was strongly linked to mental health disorders, including depression and anxiety, as well as physical conditions such as obesity and cardiovascular disease [46,47]. Encouraging physical activity through institutional wellness programs could significantly improve both mental and physical health outcomes in this population. Alcohol consumption also emerged as a factor associated with mental health. Researchers with hazardous alcohol use demonstrated higher levels of depression and emotional dysregulation [48], reinforcing the need for programs addressing substance use in academic settings. These findings suggest that targeted interventions focusing on stress management, substance use, and lifestyle modifications could substantially enhance the well-being of researchers.

Comparative analyses with other Latin American countries provide valuable context. Researchers across the region share challenges such as resource scarcity, job instability, and publication pressure. However, institutional differences and cultural factors shape the expression and management of these stressors [49,50,51]. For instance, Colombian researchers report significant barriers due to the dominance of English in academic publishing, limiting their global participation and increasing stress [50]. Similarly, structural disparities in funding and institutional support exacerbate mental health challenges across Latin America. Collaborative, cross-national studies could identify best practices for mitigating these issues and fostering healthier academic environments [49,50,51].

### 4.1. Theoretical Implications

This study contributes to the theoretical understanding of mental health among researchers by contextualizing it within Karasek’s demand–control model and emotional regulation theories. The significant associations observed between job demands, emotional dysregulation, and mental health outcomes highlight the applicability of these frameworks in academic settings. Additionally, the protective effect of advanced academic qualifications against anxiety suggests a potential interaction between professional stability and psychological resilience that warrants further exploration. These findings emphasize the need to refine theoretical models to account for the unique stressors and coping mechanisms present in academic environments.

### 4.2. Practical Implications

To address the challenges identified, institutions and policymakers must implement multifaceted strategies aimed at improving researchers’ mental health and work environments. First, organizations should establish robust mental health programs that include counseling, psychotherapy, and access to stress management workshops. Tailored approaches, such as mindfulness-based stress reduction (MBSR) programs, have demonstrated efficacy in improving emotional regulation and reducing anxiety and stress among academics. Promoting these interventions could mitigate the psychological burden faced by researchers and enhance productivity.

Second, addressing gender disparities in mental health outcomes requires targeted measures. Institutions should design gender-sensitive policies, including mentorship programs and peer-support networks that empower female researchers while addressing specific challenges such as work–life balance and professional inequities. Additionally, it is essential to foster inclusive environments that recognize and value contributions irrespective of gender thereby reducing stigma and stress associated with perceived inequalities.

Third, physical activity should be integrated into institutional wellness programs. Encouraging regular exercise through accessible facilities, structured fitness programs, and awareness campaigns can positively impact researchers’ mental and physical health. By prioritizing initiatives that promote physical activity, organizations can address the high rates of sedentary behavior observed in this study.

Fourth, the link between alcohol consumption and increased mental health challenges highlights the need for institutional efforts to promote healthier coping mechanisms. Offering resources for substance use education, peer counseling, and treatment options is vital for reducing the prevalence of harmful behaviors and improving overall well-being.

Finally, the precarious work conditions in Paraguay require structural reforms to reduce job instability and excessive workloads. Institutions must invest in sustainable funding mechanisms, provide career development pathways, and ensure equitable resource distribution. Special attention should be given to early-career researchers, who face heightened pressures and are more vulnerable to mental health challenges. Implementing these strategies can create healthier, more equitable research environments that support innovation and long-term professional satisfaction.

### 4.3. Limitations and Future Directions

The limitations of this cross-sectional study include the inability to establish causal relationships and the potential for social desirability bias due to the use of self-administered questionnaires. While internationally validated instruments (DASS-21, JCQ, DERS, and Grit-S) were employed, the limitations of these tools must be acknowledged. For example, DASS-21, while effective for screening, does not provide clinical diagnoses, and JCQ may not fully capture the cultural and contextual nuances specific to Paraguayan researchers. Additionally, the sample was restricted to SISNI researchers, which may not represent the entire population of Paraguayan researchers. The absence of longitudinal data limits the ability to assess changes in mental health and job demands over time.

Future studies should expand on this research by employing longitudinal designs to capture the evolution of mental health issues, incorporating mixed-methods approaches to enrich the understanding of cultural and contextual factors, and including broader samples beyond SISNI researchers to enhance generalizability. Investigating the impact of tailored interventions, such as mindfulness-based programs or job demand–control interventions, could also provide actionable insights for improving researchers’ well-being.

### 4.4. Strengths

Despite its limitations, this study represents the first comprehensive analysis in Paraguay examining the relationships between mental health, working conditions, and sociodemographic characteristics of scientific researchers. Using validated instruments and a diverse sample from multiple scientific fields and academic levels, it offers robust insights into subgroup differences within the academic sector. By focusing on emotional regulation and job demands, this research addresses gaps in the regional literature and provides a foundation for future investigations aimed at developing targeted interventions to support researchers’ well-being.

## 5. Conclusions

This study offers novel insights into the prevalence and determinants of mental health challenges among Paraguayan researchers, shedding light on an underexplored population in Latin America. By addressing the interplay between psychosocial factors, emotional regulation, and sociodemographic variables, this research contributes to advancing occupational health knowledge in the region, where systemic challenges and resource limitations are common.

The findings emphasize the urgent need for institutions to prioritize mental health and well-being as critical components of academic sustainability. Highlighting the role of gender disparities, job demands, and protective factors like advanced academic qualifications, this study provides a foundation for the development of regionally tailored policies and interventions.

Beyond Paraguay, this study underlines the value of cross-national collaborations in identifying best practices to mitigate workplace stress and promote equitable environments for researchers. By contextualizing these findings within Latin America, it advances the discourse on how institutional and cultural factors shape mental health outcomes, ultimately paving the way for further research and action in the field.

## Figures and Tables

**Table 1:** Participants’ sociodemographic characteristics (N = 235).

Characteristics		*n*	%
Gender	Female	126	53.6
	Male	109	46.4
Social status	Married/partnered	147	62.6
	Divorced/separated	17	7.2
	Single	70	29.8
	Widowed	1	0.4
Living arrangements	Friends/classmates	6	2.6
	Family/partner	203	86.4
	Alone	26	11.1
Children	No	92	39.1
	Yes	143	60.9

**Table 2:** Participants’ academic characteristics (N = 235).

Characteristics		*n*	%
Scientific field	Social Sciences and Humanities	75	31.9
	Medical and Health Sciences	54	23.0
	Natural Sciences, including Physics, Chemistry, and Biology	41	17.4
	Agricultural and Veterinary Sciences	33	14.0
	Engineering and Technology, including Computer Science and Mathematics	32	13.6
Type of institution	Private	58	24.7
	Public	177	75.3
Educational level	Doctorate	114	48.5
	Master’s degree	107	45.5
	Bachelor’s degree	14	6.0
SISNI * category	Beginner	118	50.2
	Level I	79	33.6
	Level II	32	13.6
	Level III	6	2.6

* SISNI = Paraguay National System of Researchers.

**Table 3:** Participants’ behavioral and health characteristics (N = 235).

Characteristics		*n*	%
Regular physical activity	≥150 min of moderate-intensity activity	72	30.6
	<150 min of moderate-intensity activity	96	40.9
	No regular physical activity	67	28.5
Behavioral habits	Tobacco use, yes	11	4.7
	Use of e-cigarettes, yes	3	1.3
	Alcohol consumption, yes	111	47.2
	Substance use, yes	8	3.4
Self-reported mental health condition	Yes	12	5.1
Diagnostic group (*n* = 12; multiple responses)	Bipolar disorders	1	8.3
	Anxiety spectrum disorders (e.g., panic, generalized anxiety)	6	50.0
	Depressive disorders (e.g., major depressive disorder)	4	33.3
	Trauma- and stressor-related disorders (e.g., post-traumatic stress disorder)	1	8.3
Type of therapy received (*n* = 12; multiple responses)	Pharmacotherapy	3	25.0
	Psychotherapy	6	50.0
	Both	2	16.6
	None	1	8.3

**Table 4:** Participants’ mental health assessments (N = 235).

Mental Health Assessments		Total (N = 235)		Females (*n* = 126)		Males (*n* = 109)	
		*n*	%	*n*	%	*n*	%
DASS 21 Depression subscale	Extremely severe depression	8	3.4	6	4.8	2	1.8
	Severe depression	8	3.4	5	4.0	3	2.8
	Moderate depression	21	8.9	12	9.5	9	8.2
	Mild depression	25	10.6	16	12.7	9	8.2
	No depression	173	73.7	87	69.0	86	79.0
DASS-21 Anxiety subscale	Extremely severe anxiety	15	6.4	11	8.7	4	3.7
	Severe anxiety	22	9.4	16	12.7	6	5.5
	Moderate anxiety	17	7.2	12	9.5	5	4.6
	Mild anxiety	18	7.7	9	7.1	9	8.2
	No anxiety	163	69.4	78	62.0	85	78.0
DASS-21 Stress subscale	Extremely severe stress	5	2.1	4	3.1	1	0.9
	Severe stress	26	11.1	17	13.5	9	8.2
	Moderate stress	20	8.5	15	12.0	5	4.6
	Mild stress	25	10.6	13	10.3	12	11.0
	No stress	159	67.7	77	61.1	82	75.3
Screened positive for alcohol-related problems (MULTICAGE CAD-4 alcohol subscale ≥ 2)	Yes	21	8.9	8	6.3	13	12.0
	No	214	91.1	118	93.7	96	88.0
Screened positive for drug-related problems (MULTICAGE CAD-4 drug subscale ≥ 2)	Yes	2	0.8	0	0.0	2	1.8
	No	233	99.2	126	100.0	107	98.2

## Data Availability

The data that support the findings of this study are available from the corresponding author upon reasonable request.
